# Phytochemical analysis and antidiabetic potential of *Elaeagnus umbellata* (Thunb.) in streptozotocin-induced diabetic rats: pharmacological and computational approach

**DOI:** 10.1186/s12906-018-2381-8

**Published:** 2018-12-13

**Authors:** Nausheen Nazir, Muhammad Zahoor, Mohammad Nisar, Imran Khan, Nasiara Karim, Heba Abdel-Halim, Akhtar Ali

**Affiliations:** 1 Department of Chemistry, University of Malakand, Chakdara Dir (L), Khyber Pakhtunkhwa Pakistan; 2 Department of Botany, University of Malakand, Chakdara Dir (L), Khyber Pakhtunkhwa Pakistan; 3 Department of Pharmacy, University of Swabi, Swabi, Khyber Pakhtunkhwa Pakistan; 4 Department of Pharmacy, University of Malakand, Chakdara Dir (L), Khyber Pakhtunkhwa Pakistan; 5 Faculty of Pharmacy and Medical Sciences, University of Petra, Amman, 11196 Jordan; 6 Global Research Laboratory, Department of BioSciences, and Engineering, Konkuk University Seoul, Seoul, South Korea

**Keywords:** HPLC, DPPH, ABTS, Type 2 diabetes, Molecular docking

## Abstract

**Background:**

The fruit of *Elaeagnus umbellata* has high medicinal values and is an excellent source of phytochemicals. This study was aimed to evaluate the antioxidant, enzyme inhibitory and antidiabetic potential of *Elaeagnus umbellata*.

**Methods:**

The antioxidant potential of the crude extract and subfractions of *E. umbellata* fruit were determined using DPPH (2, 20-diphenyl-1-picrylhydrazyl) and ABTS (2, 2′-azinobis-3-ethylbenzothiazoline-6-sulfonic acid) assays. The enzyme inhibitory potentials of extracts against α-amylase and α-glucosidase enzymes were also determined. The in vivo anti-hyperglycemic effects of the extract in STZ-induced type 2 diabetes were determined using *Sprague Dawley* adult rats. HPLC system (Agilent 1260) was used for the identification of bioactive compounds present in extracts. Molecular docking was used to identify and compare the interaction between the compounds (active constituents) and standard inhibitor acarbose with the α-amylase and α-glucosidase active sites.

**Results:**

The chloroform, ethyl acetate, and butanol fractions showed significant antioxidant potential with IC_50_ values of 40, 45 and 60 μg/mL against DPPH and 57, 70 and 120 μg/mL against ABTS free radicals respectively. The chloroform and ethyl acetate were highly active against α-amylase and α-glucosidase (IC_50_ values 58 and 200 μg/ml against α-amylase 60 and 140 μg/ml against α-glucosidase. The crude extract, chloroform, and ethyl acetate fractions were more potent in controlling the hyperglycemia in STZ-induced type 2 diabetes in rats and considerable reduction of glucose level was observed compared to the non-treated group. Furthermore, the extracts were also found useful in controlling the secondary complications associated with type 2 diabetes mellitus which was evident from the observed substantial reduction in the blood level of serum glutamate oxaloacetate transaminase, serum glutamate pyruvate transaminase, alkaline phosphatase, total cholesterol, low-density lipoproteins, and triglycerides. The molecular docking approach indicated the favorable inhibitory interaction between the docked compounds and the active sites of the α-amylase and α-glucosidase. All docked compounds occupied the same binding site as occupied by acarbose.

**Conclusion:**

It was concluded that *E. umbellata* can be used in the treatment of type 2 diabetes and oxidative stress. The extracts were also found to be effective in relieving the secondary complications associated with type 2 diabetes.

**Graphical abstract:**

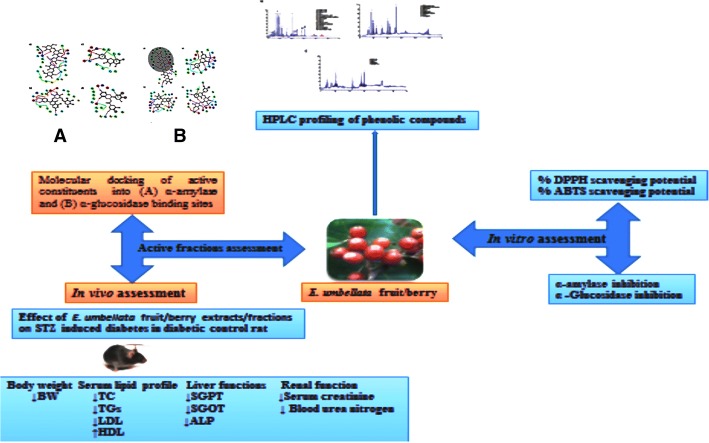

**Electronic supplementary material:**

The online version of this article (10.1186/s12906-018-2381-8) contains supplementary material, which is available to authorized users.

## Background

Reactive oxygen species (ROS) produced during metabolism of aerobic cells can damage bio-molecules like proteins, lipids, enzymes, DNA and RNA. They are involved in the progression of different chronic diseases like cancer, atherosclerosis, cardiovascular diseases, diabetes mellitus, rheumatism, nephritis, ischemic, Alzheimer’s disease (AD) and Parkinson^’^s neurodegenerative disease [1, 2]. Diabetes mellitus (DM) is a chronic metabolic disorder characterized by hyperglycemia and impaired carbohydrates, lipids and proteins metabolism [3, 4]. In diabetes mellitus either insufficient amounts of insulin is secreted by pancreatic islet cells of Langerhans or there is insulin resistance leading to an increase in blood glucose level [5]. It is a major chronic disease in human after cancer and cardiovascular diseases [6].

About 90% of diabetes cases are non-insulin dependent and are known as Type 2 diabetes (T2D) which is more prevalent in the adult’s age (≤30 years) [7]. T2D is characterized by hyperglycemia if carbohydrate-rich food is taken by the patient [8]. A number of other complications are also associated with T2D like insulin resistance, reduced insulin secretion, hyperinsulinemia, low insulin-mediated uptake and consumption of glucose [9]. T2D is a major health concern these days especially in developing and underdeveloped countries. According to the World Health Organization (WHO), approximately 350 million people are suffering from diabetes around the globe. The increases in prevalence of type 2 diabetes (T2D) is on the rise and it will become an epidemic in the near future if stringent preventive measures are not taken [10, 11].

Synthetic drugs are associated with a number of side effects. The medicines originated from plants are usually associated with least or no side effects and thus drawing an increasing attention in almost all human communities on earth [12]. All over the world attempts have been made to isolate phytoconstituents from plants having a broader range of biological activities [13]. According to Kirithikar and Basu around 800 plants have been reported having antidiabetic action with no documented side effects and minor toxicities as compared to synthetic drugs [14]. However, the phytoconstiuents responsible for their antidiabetic activity have not been fully identified. However, search for new antidiabetic drugs from plants is still attractive as they contain a number of natural compounds like glycosides, alkaloids, terpenoids, flavonoids, carotenoids which demonstrate alternative and safe effects on diabetes mellitus [15]. The easy availability, least side effects and low cost of making the herbal preparations make them the key player of all available therapies, especially in rural areas. Every plant has a natural habitat and, are restricted to particular areas on the globe. To help mankind in every part of the world, there is a need for exploring the antidiabetic potentials of unexplored plants as well [16].

*Elaeagnus umbellata* is one of the wild spiny branched shrub belongings to *Elaeagnaceae* family. It mainly grows in the Himalayan zones of Pakistan and India [17]. The *E. umbellata* fruit/berry is an excellent source of vitamins A, C, and E, minerals, flavonoids, alkaloids, steroids, terpenoids, saponins, essential fatty acids etc. [18–22]. The fruits of this plant are rich in phenolic acids (cinnamic acid and benzoic acid) and flavonoids (epigallocatechin gallate, myricetin). Furthermore, *Elaeagnus* fruits/barriers also contain a number of bioactive compounds like lutein, phytofluene, phytoene, β-carotene, β-cryptoxanthin and α-cryptoxanthin [22]. Fruit provides a number of essential components to our bodies and are helpful in the prevention of various chronic diseases including T2D. Several epidemiological studies have revealed that there is an inverse relationship between berry fruit and T2D [23, 24].

Keeping in view the high medicinal and nutritional value of *E. umbellata*, the current study was aimed to determine the antioxidant, enzyme inhibitory and antidiabetic potential of the crude extract and its subfractions. Furthermore, the phytochemicals present in the extracts of the plant were identified through HPLC-UV analysis and the active constituents were correlated with the observed biological activity through molecular docking.

## Methods

### Chemicals

DPPH: Sigma-Aldrich, CHEMIE GmbH (USA) and ABTS: Sigma-Aldrich (Germany) was used for antioxidant assays. Ascorbic acid: Sigma-Aldrich (USA); Type I α-Glucosidase (Baker Yeast); Type VI α-amylase (porcine pancreas); PNPG (*p*-nitrophenyl-α-D-glucopyranose): Sigma-Aldrich (Paris, France). Streptozotocin (STZ) from Sigma Aldrich (Germany); Glucose estimation kits from S.D. Chek-Gold (Germany) and glibenclamide from Sanofi-Aventis-Pharma (Pakistan) was used for the antidiabetic study. Solvents like methanol, *n*-butanol*, n*-hexane, ethyl acetate, and chloroform: Merck (Germany); Tween-80 from Scharlau-chem. (Spain); normal saline solution from Utsoka Pharma (Pakistan); Lipid profile tests kits from Human (Germany) and Renal profile tests kits from Bioneed (Germany; diagnostic). Analytical grade chemicals were used in this study.

### Plant material and sample preparation

The fruits of *E. umbellata* Thunb. has shown in (Additional file 1: Figure S1) were collected from the hilly areas of Kalam, Malakand Division, Khyber Pakhtunkhwa, Pakistan in August–September 2016. The plant sample was identified by plant taxonomist; Prof. Mehboob-UR-Rahman, PGC. Swat, Khyber Pakhtunkhwa, Pakistan. The plant specimens were deposited in the Botanical Garden Herbarium, University of Malakand, Pakistan with voucher number BGH.UOM.154. The berries were cleaned and kept on a clean paper to dry in shade for 20 days.

The dried fruits (10 kg) were crushed through a grinder before maceration in 80% methanol. The resulting mixture was kept for 14 days with periodical shaking and was then filtered through muslin cloth followed by filtration with Whattman filter paper. The filtrates were converted into semisolid mass under reduced pressure at 40 °C in the rotary evaporator (Schwabach: 4000; Heidolph-Laborota-Germany). The semisolid mass obtained was solidified in open air (final mass = 750 g). The crude extract was subjected to fractionation by solvent-solvent extraction method. A specified amount of crude extract was dissolved 500 mL distilled water in a separating funnel and partitioned with different solvents starting from a low to high polarity (*n*-hexane, chloroform, ethyl acetate, and *n*-butanol). About 95, 210, 115, 90 and 220 g solid extracts were obtained from *n*-hexane, chloroform, ethyl acetate, *n*-butanol, and aqueous fractions respectively after evaporation.

### Extracts preparation for HPLC-UV characterization

About 1 g powdered berry sample was mixed in methanol and water mixture (1:1; 20 mL; *v*/v). The mixture was heated at 70 °C for 1 h in a water bath and centrifuged for 10 min at 4000 rpm. The supernatants (2 mL) were then filtered through Whatman filter paper into HPLC vials.

For the identification of phenolic compounds, the High-performance liquid chromatography (HPLC) Agilent-1260 infinity system was used. The separation was achieved using Agilent-Zorbax-Eclipse column C18. Column gradients system was consist of solvent B (deionized water: methanol: acetic acid in the ratio of 180: 100: 20; *v*/v) and solvent C (deionized water: methanol: acetic acid in the ratio of 80: 900: 20; v/v). The gradient system was started with solvent B 100, 85, 50 and 30% at 0, 5, 20 and at 25 min, and finally, solvent C (100%) started from 30 to 40 min. Identification of phenolic compounds were made by comparing the retention times of corresponding component in the HPLC chromatogram with that of the available standards chromatogram while quantification of the compounds were done through single point calibration, taking into consideration the percent peak area [25].

### Antioxidant scavenging assays

#### DPPH scavenging assay

For the determination of DPPH (2, 20-diphenyl-1-picrylhydrazyl) free radical scavenging ability of the extracts, Brand-Williams assay [26] was used with some modification. About 24 mg DPPH was dissolved in 100 mL methanol. Plant sample stock solutions (1 mg/mL) were also prepared in methanol. Using serial dilutions working solutions with the following concentrations: 1000, 500, 250, 125, 62.5 and 31.05 μg/mL were prepared. About 0.1 mL of each working dilution was mixed with DPPH (3.0 mL) and incubated at 23 °C for 30 min. Absorbance was measured at 517 nm via UV-spectrophotometer (Thermo Electron Corporation: USA). Ascorbic acid was used as a standard. Results were presented as Mean ± SEM. % DPPH scavenging potential was calculated by the following formula:

1 $$ \% DPPH\ Scavenging\ potential=\frac{\  control\ absorbance- sample\ absorbance}{control\ absorbance}\times 100 $$

#### ABTS scavenging assay

Antioxidant potential of berry extracts were also determined against ABTS (2, 2′-azinobis-3-ethylbenzothiazoline-6-sulfonic acid) free radical by method described by Re et al. [27]. ABTS (7 mM) and potassium persulfate (2.45 mM) solutions were mixed thoroughly and were incubated overnight in dark for the production of ABTS free radical. The absorption of this mixture was adjusted by adding methanol to 0.7 at 745 nm. About 300 μL extract working dilutions and 3.0 mL ABTS solutions were mixed and incubated for 6 min. Finally the absorbance was measured via UV spectrophotometer. Ascorbic acid was used as positive control. % ABTS scavenging potential was calculated using the following formula:

2 $$ \% ABTS\ Scavenging\ potential=\frac{control\ abso rbance- sample\ abso rbance}{control\ abso\mathrm{r} bance\ }X100 $$

#### In vitro α-amylase enzyme inhibition

The extracts solutions were prepared in normal saline with Tween-80 (5%) using the reported method [28]. The α-amylase enzyme inhibition potential was evaluated using 3, 5-dinitrosalicylic acid (DNSA) assay [29]. The Me-Ext and subsequent fractions of *E. umbellata* were dissolved in DMSO (10%), 0.02 M Na_2_HPO_4_/NaH_2_PO_4_ buffer and 0.006 M NaCl at pH 6.9. Through serial dilutions the working solutions; 31.05, 62.5, 125, 250, 500 and 1000 μg/mL were prepared. 200 μl of α-amylase (2 units/ml) solution was mixed with working dilutions (200 μl) and incubated at 30 °C for 10 min. Subsequently 200 μl starch (1% in water: (*w*/*v*)) solution was added to each sample dilution followed by incubation for 3 min. The reaction was stopped by adding of,200 μl sodium potassium tartrate tetrahydrate (DNSA) reagent (12 g) dissolve in 8.0 mL, 2 M NaOH and 20 mL of 96 mM 3, 5 dinitrosalicylic acid solution. The reaction mixture was boiled for 10 min in a water bath at 85–90 °C. After cooling, dilution was done with 5 mL distilled water and finally, the absorbance was noted at 540 nm. A blank solution was prepared containing only plant extract but no enzyme. Standard acarbose (100 μg/ml–2 μg/ml) was used as positive control (without plant extract). The α-amylase enzyme inhibitory potential was calculated by the following formula:

3 $$ \%\alpha - amylase\ Inhibition=\frac{control\ absorbance- sample\ absorbance}{control\ absorbance}X100 $$

#### In vitro α –glucosidase enzyme inhibitory assay

The α-glucosidase inhibition by Me-Ext and subsequent fractions were carried out according to the reported method of Ranilla et al. [30] with minor changes. The reaction mixture was formulated by adding 100 μl α-glucosidase enzyme (0.5 unit/ml), 0.1 M phosphate buffer (600 μl) at pH 6.9 and 50 μl each sample dilutions (31.05, 62.5, 125, 250, 500 and 1000 μg/mL). The mixture was incubated for 15 min at 37 °C. The enzymatic reaction was started by adding 100 μl *p*-nitro-phenyl-α-D-glucopyranoside (5 mM) solution in 0.1 M phosphate buffer at pH 6.9 followed by 15 min incubation at 37 °C. The reaction was stopped by adding 400 μl sodium carbonate (0.2 M) solution. The absorbance of the final reaction mixture was recorded at 405 nm. The reaction mixture with no plant extract was used as positive control while the blank solution was prepared without enzyme α-glucosidase. The α-glucosidase % inhibition was calculated using formula:

4 $$ \%\alpha - Glucosidase\ Inhibi\mathrm{t} ion=\frac{control\ absorbance- sample\ absorbance}{control\ absorbance}\times 100 $$

### Animals

*Sprague Dawley* adult rats (150 to 170 g body weight) were purchased from Rifah Institute of Pharmaceutical Sciences Islamabad. Animal’s acclimatization was carried out for 1 week in the laboratory animal house. The animals were provided with standard food as ad libitum fresh water. The animals were kept at room temperature around 22–25 °C with light and dark cycle of about 12 h each. All procedures related to the animals were carried out according to the Animal Scientific Procedure Act; UK (1986) and approval was taken from the Departmental Animal Ethical Committee (DAEC/PHARM/2016/1) of University of Swabi.

### Acute toxicity study of the fruit Me-Ext/fractions of *Elaeagnus umbellata* Thunb.

The acute toxicity of the Me-Ext/fractions of *E. umbellata* were evaluated according to the protocol described by Karim et al. [31] using adults *Sprague Dawley* rats weighing 150–200 g. All animals were divided into seven groups and each group composed of 8 animals. The control group animals received tween-80 suspension, orally. All animals were then treated orally with different doses of extract/fractions 100, 200, 400, 500, 1000, 1500 and 2000 mg/kg. After administration, the animals were observed for 0, 0.5, 1.0, 24, 48, 72 and 168 h for physical, behavioral and pharmacological lethal effects. The extracts did not produce any drug-induced harmful physical signs and no mortality was detected. The extract remained safe and nontoxic up to 2000 mg/kg dose range. Therefore, according to OECD guidelines, 200 mg/kg extract dose that is 1/10th of 2000 mg/kg dose (maximum tested dose) was selected to evaluate the in vivo antidiabetic activity [32]. All the doses of the extracts/fractions (200 mg/kg) were made by dissolving it in tween-80 suspension and standard glibenclamide drug (0.5 mg/kg, p.o) in normal saline and were administered orally.

### Animal experimental design for inducing type 2 diabetes

T2D was induced according to the method previously described by Gopalakrishnan et al. [33]. Animals were divided into two major groups. One group was given normal pellet diet and the other animal group was fed with high fat diet (HFD) (40% raw beef fat + 30% casein + 10% glucose + 7% wheat flour + 6% barn + 4% vitamin mixture and 3% salt mixture) for 2 weeks before commencing the experiment. After 2 weeks, induction of hyperglycemia was carried out in HFD *Sprague Dawley* rats via a single intraperitoneal (*i.p*) injection of STZ (50 mg/kg) prepared in 0.9% normal saline solution after an overnight fast. Subsequently 72 h after administration of STZ, blood samples were collected from the tail vein via Glucometer strips by means of SD glucometer (Germany) and blood glucose level was measured [34]. Rats having fasting blood glucose level ≥ 300 mg/dl were considered hyperglycemic and were included in the study (Table 1).

**Table 1:** Experimental design and various tretament groups used in the study

Group	Group Category	Treatment given	Route
Group I	Normal control	Normal saline 8 mL/kg	p.o.
Group II	Diabetic control	STZ (50 mg/kg)	i.p.
Group III	Positive control	Glibenclamide 0.5 mg/kg	p.o
Group IV	Me-Ext	100 mg/kg	p.o
Group V	Me-Ext	200 mg/kg	p.o
Group VI	Chf-Ext	100 mg/kg	p.o
Group VII	Chf-Ext	200 mg/kg	p.o
Group VIII	EtAc-Ext	100 mg/kg	p.o
Group IX	EtAc-Ext	200 mg/kg	p.o

*Me-Ext* Methanolic extract, *Chf-Ext* Chloroform extract fraction, *EtAc-Ext* Ethyl acetate extract fraction, *STZ* Streptozotocin, *p.o.* Per oral, *i.p.* Intraperitoneal

### Treatment protocol

Rats were divided into 9 groups (n=8) after an overnight fast for about 12 h. The first group was labelled as a normal control and was given normal saline orally while the rest of the eight groups were considered HFD groups. The second group categorized as diabetic control and was given normal saline. Standard 0.5 mg/kg glibenclamide drug (p.o) was given to the third group. The fourth group received the crude Me-Ext of *E. umbellata* (100 and 200 mg/kg; p.o), while the fifth, sixth, seventh, eighth and ninth groups were given Chf-Ext and EtAc-Ext fraction of *E. umbellata* (100 and 200 mg/kg; p.o), respectively.

The treatment of plant extracts was continued for 21 days (daily at 09:00 am). The level of blood glucose and body weights were measured on 0, 4th, 7th, 10th, 15th, 21st day of treatment according to the previous protocol described by Bhat et al. [35].

### Collection of blood and estimation of biochemical parameters

At the completion of in vivo antidiabetic activity on 21st day, all animals were anesthetized via 35 mg/kg pentobarbital sodium and euthanized by cervical decapitation using previous procedure illustrated in schedule-1 of UK, animal scientific procedure act; 1986. Blood collection was carried out via cardiac puncture for studying the biochemical parameters [35]. The blood samples were centrifuged for serum separation at 3500 rpm (Centurion scientific Pvt., Ltd. UK) for 10 min. The serum was analyzed through spectrophotometer (Perkin Elmer; Germany) for investigation of biochemical parameters like serum glutamate pyruvate transaminase (SGPT), serum glutamate oxaloacetate transaminase (SGOT) and serum alkaline phosphatase (ALP). Total cholesterol (TC), Triglycerides (TG), Low-density lipoproteins (LDL), High-density lipoprotein (HDL) and serum creatinine were measured by CHOD-PAP and GPO-PAP procedure (Human kit; Germany) using UV-Spectrophotometer [36].

### Molecular docking

**Fig. 1:**
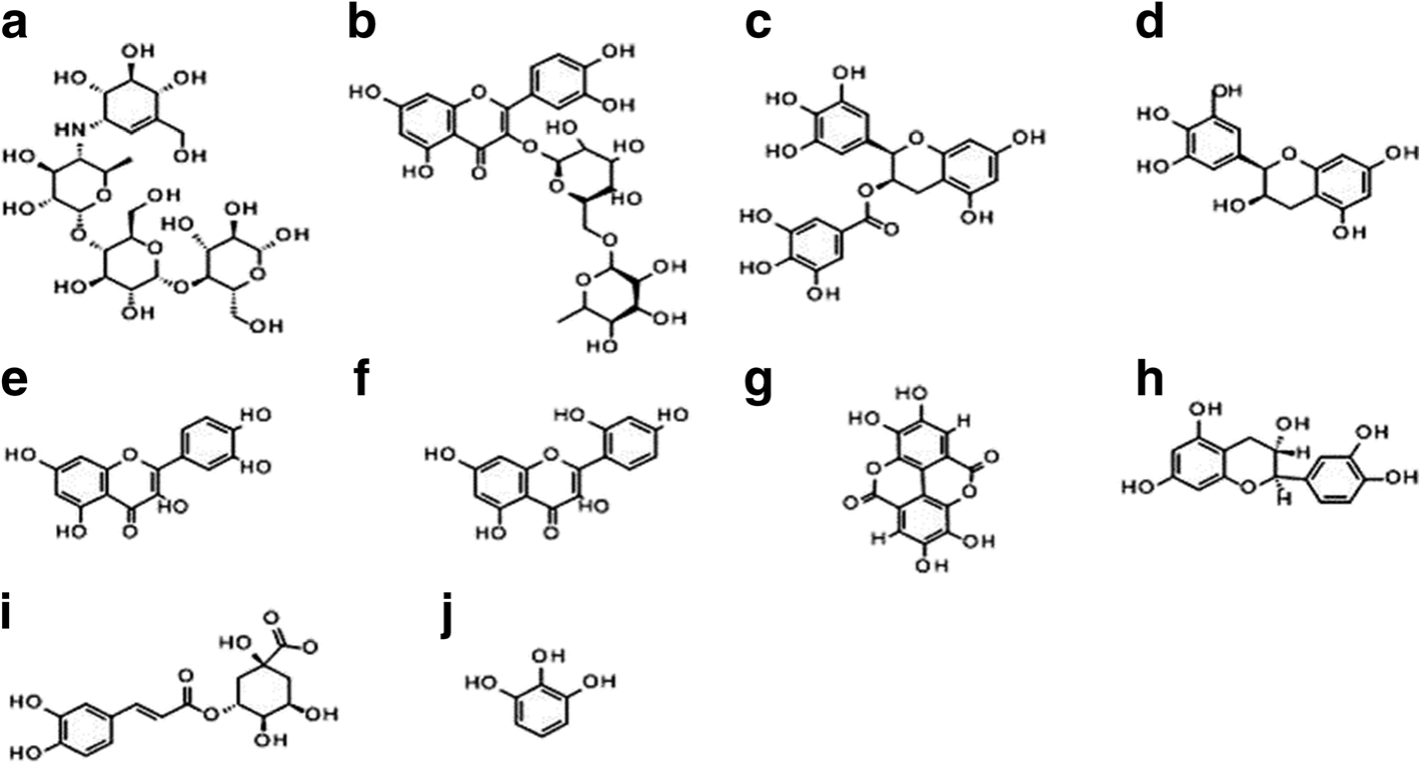
Phenolic Compounds identified in *E. umbellata* Thunb. fruit methanolic extract/fractions studied in molecular docking. **a** Acarbose, **b** Rutin, **c** Epigallocatechin gallate, **d** Epigallocatechin, **e** Quercetin, **f** Morin, **g** Ellagic acid, **h** Catechin, **i** Chlorogenic acid, **j** Pyrogallol

The X-ray crystal structure of α-glucosidase (PDB code: 2ZE0, 2 Å resolution) [37] and the crystal structure of α-amylase complexed with acarbose was downloaded from Protein Data Bank (PDB code: 3BAJ, 2.1 Å resolution) [38]. The two protein structures were organized in the Schrödinger software with the protein preparation module. Inside the structures, water molecules were removed [39]. The two structures were subjected to subsequent preparation stages: addition of hydrogen, assigning of protonation state and partial charges. Finally, the protein structures were minimized using the OPLS force field in the Macro-Model module. The minimization was achieved up to the average root-mean-square deviation of all the non-hydrogen atoms extended 0.3 Å. Selection of ligands used in docking were based on the natural constituents found in the Chf-Ext and EtAc-Ext layers: quercetin, rutin, chlorogenic acid, epigallocatechin gallate, morin, catechin hydrate, pyrogallol, ellagic acid, gallic acid. To validate our docking results acarbose and epigallocatechin were added to the list (Fig. 1).

All compounds were built using the fragment library (Maestro; 10.6) and were set via Lig-Prep module. Optimization of ligands was carried out by an OPLS-2005 force field in the Macro-Model module [40].

The docking procedure for α-amylase created the production of a grid box and the docking site was designated as the centroid of the acarbose molecule. However, as the α-glucosidase enzyme is crystallized without any ligand, the binding site was determined using Sitemap [41] and the grid generation proceeded by a grid box formation that is the centroid of the amino acids surrounding this binding site Arg407, Asp326, Arg197, and Asn258.

For both α-glucosidase and α-amylase enzymes, the defaulting grid size was taken from the Glide program [42]. Consequently, the docking of ligands was occurred into the definite binding site by means of Grid-Based docking and flexible glide docking (Glide-XP) using the default parameters of docking with no constraints. Docking of Ligands occurred into the stiff receptor lacking ligand nonpolar atoms or scaling-down the Vander Waals radii of receptor atoms.

The best-docked structures that have more favorable binding were selected using the Glide-Score function with more negative Glide-Score. After visualization of the ligand-protein complex, the interactions were studied among different ligand-receptor.

### Statistical analysis

The IC_50_ values were measured by linear regression analysis among the % DPPH and ABTS free radical scavenging potentials by different concentrations of test samples using Excel program. Regression (*y*) and linear correlation (R^2^) were used to determine the antioxidant and enzyme inhibition potentials of samples using Excel 2007. All in vivo experiments were performed in three replicates. The results were presented as Mean ± SEM and Student’s t-test and one way ANOVA followed by Dunnett’s posthoc multiple comparison test used to determine the values of P. *P* < 0.05 were considered as significant.

## Results

### Identification of phenolic compounds through HPLC-UV technique

**Fig. 2:**
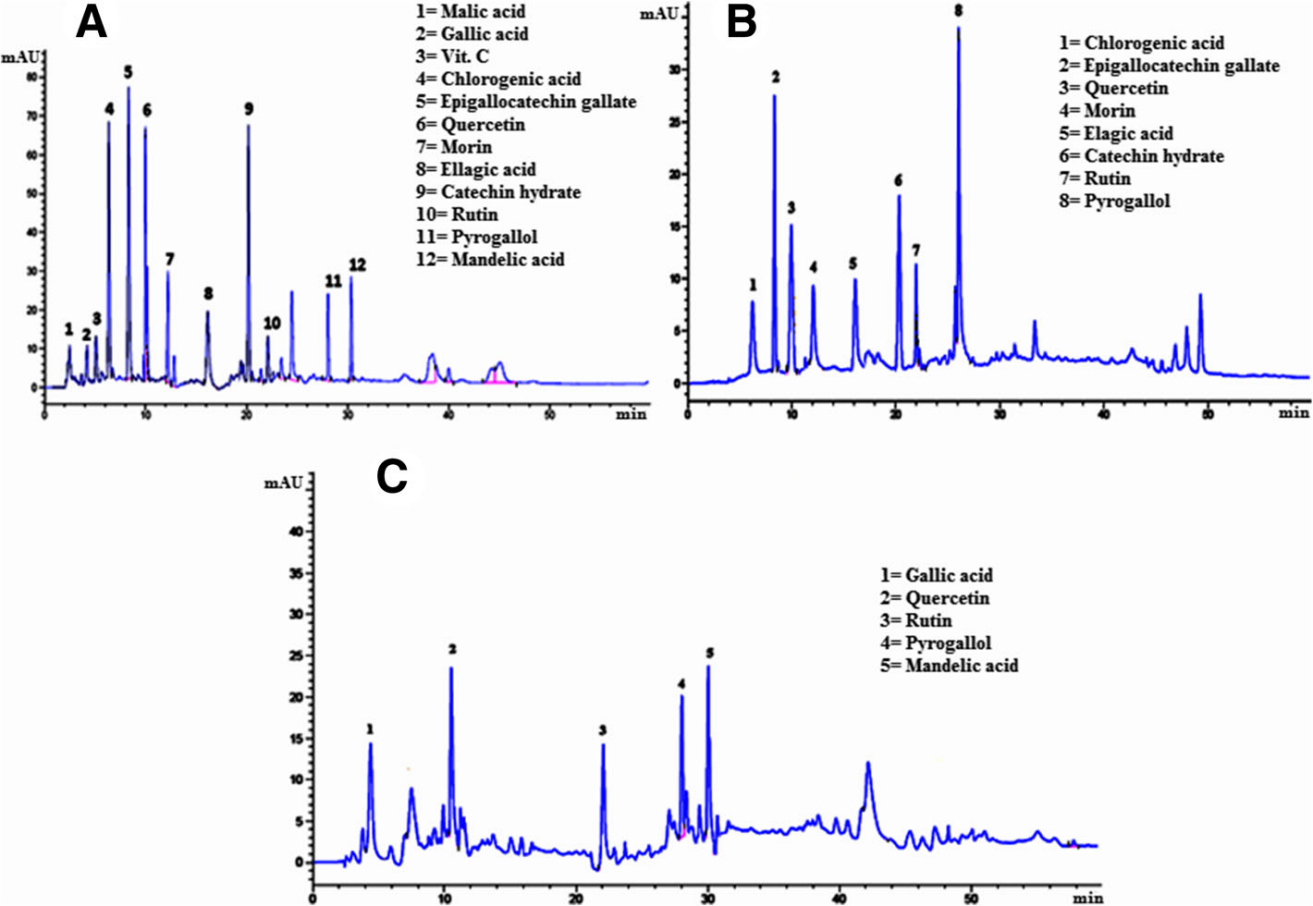
HPLC-UV Chromatograms of phenolic compounds in *E. umbellata* Thunb. fruit **a** Me.Ext, **b** Chf-Ext and **c** EtAc-Ext

Typical HPL-UV chromatograms of *E. umbellata* fruit Me-Ext/fractions are presented in Fig. 2. A total of 12 phenolic compounds (malic acid, gallic acid, vitamin C, chlorogenic acid, epigallocatechin gallate, quercetin, morin, ellagic acid, catechin hydrate, rutin, pyrogallol and mandelic acid) were identified in the Me-Ext while eight phenolic compounds including chlorogenic acid, epigallocatechin gallate, quercetin, morin, ellagic acid, catechin hydrate, rutin, and pyrogallol were identified in the Chf-Ext. In EtAc-Ext five phenolic compounds (gallic acid, quercetin, rutin, pyrogallol, and mandelic acid) were identified (Fig. 2). The Quantification and identification of each phenolic compound with their particular peak position and retention time (Rt) in the chromatogram is presented in Table 2. All these phenolic compounds were identified with standard phenolic compounds in fruit samples of *E. umbellata*. Quantification of antioxidants was carried out by using the formula:

5 $$ Cx=\frac{Ax\times Cs\left(\raisebox{1ex}{$\upmu \mathrm{g}$}\!\left/ \!\raisebox{-1ex}{$ ml$}\right.\right)\times V(ml)}{As\times Sa\mathrm{m} ple\ \left( wt. in\ g\right)} $$

**Table 2:** Identification and Quantification of phenolic compounds in *E. umbellata* Thunb. fruit Me-Ext/fractions

Sample Extract	No. of Peak	Retention time (min)	Phenolic compounds Identity	HPLC-UV λmax (nm)	Peak Area of sample	Peak Area of standard	Concentration (μg/ml)	Identification Reference
Me.Ext	1	2.7	Malic acid	320	434.5	40.3	9.7	Standard
	2	4.3	Gallic acid	320	25.3	195.4	0.1	Standard
	3	4.6	Vitamin C	320	18.2	22.4	0.7	Standard
	4	6.0	Chlorogenic acid	320	331.6	12.9	23.1	Standard
	5	8.9	Epigallocatechin gallate	320	972.0	72.6	12.1	Standard
	6	10.3	Quercetin	320	1849.2	90.9	18.3	Standard
	7	12.3	Morin	320	25.7	2.0	11.5	Standard
	8	16.7	Elagic acid	320	36.4	319.2	0.1	Standard
	9	20.0	Catechin hydrate	320	226.5	78.0	2.6	Standard
	10	22.7	Rutin	320	69.4	22.4	2.8	Standard
	11	28.1	Pyrogallol	320	11.8	1.0	10.5	Standard
	12	30.4	Mandelic acid	320	34.2	72.0	0.4	Standard
Chf-Ext	1	6.0	Chlorogenic acid	320	126.5	12.9	8.8	Standard
	2	8.9	Epigallocatechin gallate	320	4706.1	7261.5	58.3	Standard
	3	10.3	Quercetin	320	899.1	9089.3	8.9	Standard
	4	12.3	Morin	320	63.6	11.5	5.0	Standard
	5	16.7	Elagic acid	320	148.0	319.2	0.4	Standard
	6	20.0	Catechin hydrate	320	3449.0	78.0	39.8	Standard
	7	22.7	Rutin	320	1126.2	2241.2	45.2	Standard
	8	28.1	Pyrogallol	320	58.5	1.0	52.1	Standard
EtAc-Ext	1	4.3	Gallic acid	320	966.3	195.4	4.5	Standard
	2	10.3	Quercetin	320	1302.0	90.9	12.9	Standard
	3	22.7	Rutin	320	355.0	22.4	14.3	Standard
	4	28.1	Pyrogallol	320	53.3	1.0	47.5	Standard
	5	30.4	Mandelic acid	320	488.7	72.0	6.1	Standard

***Cx*** = Sample concentration; ***As*** = Standard peak area; ***Ax*** = Sample peak area; ***Cs*** = Standard concentration (0.09 μg/ml).

### DPPH (2, 20-diphenyl-1-picrylhydrazyl) scavenging potential

**Fig. 3:**
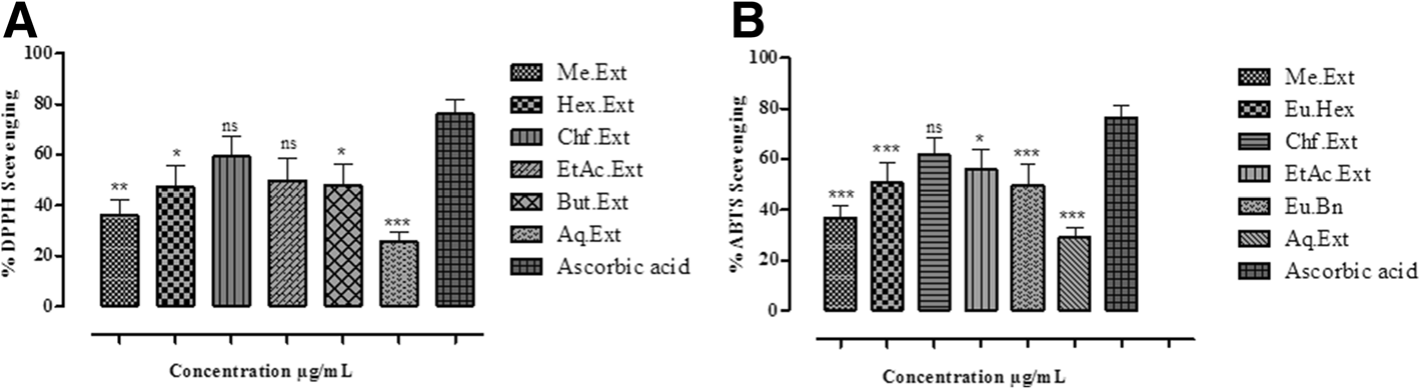
**a** % DPPH **b** ABTS Scavenging activity of Me-Ext and subsequent fractions of *E. umbellata* fruit at various concentrations. The data is represented as Mean ± SEM, *n* = 3. Values are significantly different as compared to positive control (*P< 0.05, **P< 0.01, ***P< 0.001)

The crude Me-Ext, Hex-Ext, Chf-Ext, EtAc-Ext, But-Ext and Aq-Ext inhibited DPPH by 55 ± 1, 79 ± 1, 88 ± 1, 83 ± 1, 80 ± 1 and 40 ± 1% with their IC_50_ values 550, 80, 40, 45, 60 and 1300 μg/mL respectively at the maximum concentration of 1000 μg/mL. The results indicated that Chf-Ext and EtAc-Ext caused significant inhibition with the lowest IC_50_ values comparable to standard ascorbic acid (Table 3 and Fig. 3a). The standard ascorbic acid caused 95 ± 1% inhibition at 1000 μg/mL with an IC_50_ value of 30 against DPPH.

**Table 3:** DPPH and ABTS free radical Scavenging activity of *E. umbellata* Thunb. fruit Me-Ext and various fractions

S. No	Sample Extracts	IC_50_ (μg/mL) of DPPH	IC _50_ (μg/mL) of ABTS
1	Me-Ext	550	760
2	Hex-Ext	80	135
3	Chf-Ext	40	57
4	EtAc-Ext	45	70
5	But-Ext	60	120
6	Aq-Ext	1300	1175
7	Acarbose	30	32

*Me-Ext* Methanolic extract, *Hex.Ext* n-hexane extract fraction, *Chf-Ext* Chloroform extract fraction, *EtAc-Ext* Ethyl acetate extract fraction, *But.Ext* n-Butanol extract fraction, *Aq.Ext* Aqueous extract fraction, *DPPH* 2, 20-diphenyl-1-picrylhydrazyl, *ABTS* 2, 2′-azinobis-3-ethylbenzothiazoline-6-sulfonic acid, *IC*_*50*_ Median inhibitory concentration

### ABTS (2, 2′-azinobis-3-ethylbenzothiazoline-6-sulfonic acid) free radical scavenging potential

ABTS free radical scavenging of the Me-Ext and their subsequent fractions are presented in Table 3 and Fig. 3b. The % ABTS inhibition of Me-Ext, Hex-Ext, Chf-Ext, EtAc-Ext, But-Ext and Aq-Ext were 55 ± 1, 80 ± 1, 87 ± 1, 84 ± 1, 78 ± 1 and 43 ± 1 with their IC_50_ values 760, 135, 57, 70, 120 and 1175 μg/mL respectively at the maximum concentration of 1000 μg/mL. The results indicated that Chf-Ext and EtAc-Ext caused significant inhibition with lowest IC_50_ values (Table 3 and Fig. 3b). Ascorbic acid caused 91 ± 1 inhibition at 1000 μg/mL with IC_50_ value of 32 μg/mL against ABTS.

### In vitro α-amylase enzyme inhibitory assay

**Fig. 4:**
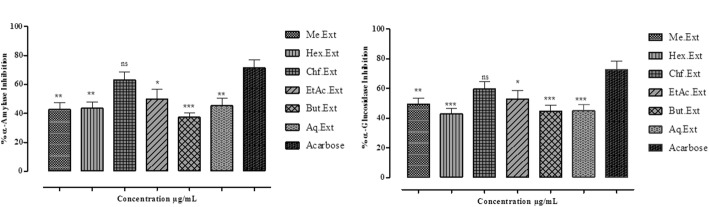
**a** % α-amylase **b** α-glucosidase inhibition potential of *E. umbellata* fruit Me-Ext and subsequent fractions at various concentrations. The data is represented as Mean ± SEM, n = 3. Values are significantly different as compared to positive control (*P< 0.05, **P< 0.01, ***P< 0.001)

The IC_50_ values were calculated by evaluating the plot of % α-amylase enzyme inhibition as a function of extract/fractions concentrations (Fig. 4a, Table 4). % α-amylase inhibition potential of Me-Ext, Hex-Ext, Chf-Ext, EtAc-Ext, But-Ext and Aq-Ext were 59 ± 1, 57 ± 1, 81 ± 1, 72 ± 1, 47 ± 0.2 and 63 ± 1 with their IC_50_ values 400, 240, 58, 200, 620 and 360 μg/mL at the highest concentration (1000 μg/mL). The Chf-Ext was found to be the most effective and potent fraction and showed the highest % α-amylase inhibition with the lowest IC_50_ value (Table 4). Acarbose was used as a standard which caused 86 ± 1% inhibition at the maximum concentration of 1000 μg/mL with IC_50_ value 30 μg/mL.

**Table 4:** α-Amylase and glucosidase inhibition potential of *E. umbellata* fruit Me-Ext and various fractions

S. No	Sample Extracts	IC_50_ (μg/mL) α-glucosidase	IC _50_ (μg/mL) α-amylase
1	Me-Ext	200	400
2	Hex-Ext	400	240
3	Chf-Ext	60	58
4	EtAc-Ext	140	200
5	But-Ext	420	620
6	Aq-Ext	240	360
7	Acarbose	30	32

*Me-Ext* Methanolic extract, *Hex.Ext* n-hexane extract fraction, *Chf-Ext* Chloroform extract fraction, *EtAc-Ext* Ethyl acetate extract fraction, *But.Ext* n-Butanol extract fraction, *Aq.Ext* Aqueous extract fraction, *IC50* Median inhibitory concentration

### In vitro α–glucosidase enzyme inhibitory assay

The IC_50_ values were determined by measuring the plot of % α-glucosidase enzyme inhibition as a function of extract/fractions concentrations (Fig. 4b, Table 4). The % α-glucosidase inhibition of Me-Ext, Hex-Ext, Chf-Ext, EtAc-Ext, But-Ext and Aq-Ext were 62 ± 1.1, 55 ± 1, 78 ± 1.0, 70 ± 1, 57 ± 1.0 and 62 ± 1.2 with their IC_50_ values 400, 60, 140, 420, 240,240 μg/mL at the highest concentration of 1000 μg/mL. The Chf-Ext was the most potent fraction and showed the highest % α-glucosidase inhibition potential with the lowest IC_50_ value (Table 4). Acarbose was used as a standard which caused 88 ± 1% inhibition at the maximum concentration (1000 μg/mL) with IC_50_ value 32 μg/mL.

### Acute toxicity study

The Me-Ext/fractions (100–2000 mg/kg) of *E. umbellata* did not produce any significant behavioral alterations (respiratory aches, convulsions shortage, writhing, variations to reflex actions or mortality) in animals. An insignificant increase in petulance was detected at 2000 mg/kg dose in three animals out of eight. All animals appeared healthy at 24 h to 1 week with no noticeable variations in appearance or behavior. No mortality has been noticed up to 1 week.

### Estimation of biochemical parameters

#### Effect of *E. umbellata* Thunb. methanolic fruit extract/fractions on glycemia

**Fig. 5:**
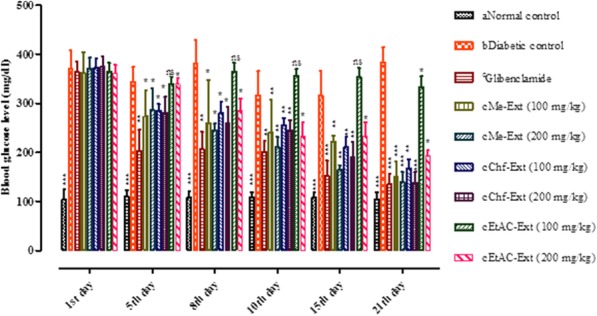
Effect of *E. umbellata* fruit methanolic extracts/fractions and glibenclamide on blood glucose level in STZ-induced diabetic rats. Each value is Mean ± SEM of 8 animals. Comparisons were made between ^a^ normal control to ^b^ diabetic control using student t-test (***P< 0.001) and between ^b^diabetic control to positive control ^c^(Glibenclamide/extracts treated groups) using one way ANOVA followed by Dunnett’s multiple comparison test (*P< 0.05, **P< 0.01, ***P< 0.001)

The effect of *E. umbellata* Me-Ext their subsequent fractions Chf-Ext, EtAc-Ext (100 and 200 mg/kg) and standard glibenclamide on variations in blood glucose in normal control group, diabetic control, and plant extracts treatment group are shown in Fig. 5 and (Additional file 2: Table S1). Oral administration of the Me-Ext and Chf-Ext (100 and 200 mg/kg) caused a significant decrease in blood glucose level compared to diabetic control at the end of 21st day treatment. Blood glucose level reduction was observable from the 5th day and onward. The EtAc-Ext did not show any significant blood glucose reduction at 100 mg/kg, however, it showed significant reduction in blood glucose at 200 mg/kg but the effect was much weaker than the Chf-Ext at the end of treatment period. Furthermore, the onset of the effect was also delayed and significant lowering in blood glucose was seen from 10th day and onward.

#### Effect *of E. umbellata* Thunb. methanolic fruit extract/fractions on body weight in diabetic rats

**Fig. 6:**
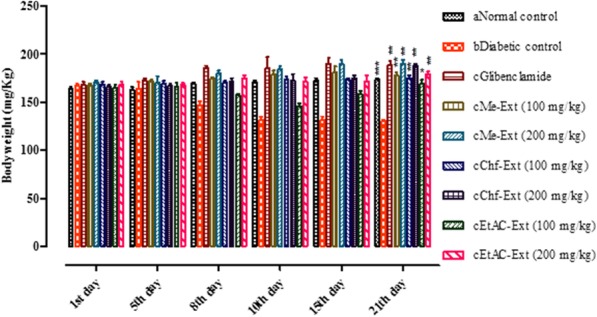
Effects of *E. umbellata* fruit methanolic extracts/fractions on body weight in STZ-induced diabetic rats. Each value is Mean ± SEM of 8 animals. Comparisons were made between anormal control to ^b^diabetic control using student t-test (***P< 0.001) and between ^b^diabetic control to positive control ^c^(Glibenclamide/extracts treated groups) using one way ANOVA followed by Dunnett’s multiple comparison test (*P< 0.05, **P< 0.01)

The effect of *E. umbellata* Me-Ext their subsequent fractions Chf-Ext, EtAc-Ext (100 and 200 mg/kg) and standard glibenclamide on changes in body weight in the normal control group, diabetic control, and plant extracts treatment group are shown in Fig. 6 and (Additional file 3: Table S2). STZ-induced diabetic rats revealed significant reduction in body weight as compared to normal control rats during the experimental study period. Loss in body weight continued in diabetic control rats till the end of 21st-day treatment. The Me-Ext, Chf-Ext, and EtAc-Ext (100 and 200 mg/kg) reversed the STZ-mediated reduction in body weight and caused significant increases in body weight at the end of 21 days treatment.

#### Measurement of serum lipid profile in diabetic rats

The levels of parameters of lipid profiles including TC, TGs, LDL, HDL and cholesterol in normal control group, diabetic control group and plant extracts treatment group are shown in Table 5. Diabetic control group showed a significant increase in TC, TGs, LDL and cholesterol while a significant decrease was observed in HDL cholesterol compared to normal control group (Table 5). The Me-Ext, Chf-Ext, and EtAc-Ext (100 and 200 mg/kg) showed a significant decrease in TC, TGs, LDL and cholesterol as compared to diabetic control group at the end of 21 days treatment. Furthermore, the Me-Ext, Chf-Ext, and EtAc-Ext (100 and 200 mg/kg) also significantly increased HDL cholesterol in diabetic rats at the end of 21 days of treatment.

**Table 5:** Effect of *E. umbellata* fruit methanolic extract/fractions on lipid profile in streptozotocin induced diabetic rats

S.No	Groups	Dose (mg/kg)	TC (mg/dl)	TGs (mg/dl)	HDL(mg/dl)	LDL(mg/dl)
1	^a^Normal control	0.3 ml	125 ± 6.10**	123.6 ± 8.5**	37 ± 3.1*	74 ± 5.5**
2	^b^Diabetic control	0.3 ml	163.3 ± 6.5	165.0 ± 7.9	25.2 ± 2.2	170.4 ± 8.9
3	^c^Glibenclamide	0.5	138.5 ± 6.3**	125.3 ± 5.5**	40.5 ± 4.5**	89.3 ± 5.5***
4	^c^Me-Ext	100	140.2 ± 5.3*	145.5 ± 7.7*	33.5 ± 4.3*	145.6 ± 4.2*
5	^c^Me-Ext	200	131.5 ± 7.5**	138.8 ± 6.5**	36.6 ± 5.5*	93.5 ± 4.6**
6	^c^Chf-Ext	100	145.4 ± 5.5*	143.2 ± 3.2*	34.2 ± 2.5*	125.3 ± 3.5*
7	^c^Chf-Ext	200	135.4 ± 5.7**	133.2 ± 5.1**	37.2 ± 3.5*	95.3 ± 3.5**
8	^c^EtAC-Ext	100	147.2 ± 5.6*	146.8 ± 4.0*	30.1 ± 4.5*	123.8 ± 6.0*
9	^c^EtAC-Ext	200	137.2 ± 7.6**	136.8 ± 5.0**	36.1 ± 5.5*	93.8 ± 8.0**

Each value is mean ± SEM of 8 animals. Comparisons were made between ^a^normal control to ^b^diabetic control using student t-test (*p < 0.05, **p < 0.01) and between ^b^diabetic control to positive control ^c^(Glibenclamide/extracts) treated groups using one way ANOVA followed by Dunnett’s posthoc multiple comparison test (* *p* < 0.05,** *p* < 0.01, ***p < 0.001)

#### Effect of *E. umbellata* Thunb. methanolic fruit extract/fractions on the liver and renal functions in STZ-induced diabetic rats

The activity of hepatic enzymes like SGPT, SGOT and ALP and renal functions like serum creatinine and blood urea nitrogen in the normal control group, diabetic control group, and plant extracts treatment group are shown in Table 6. STZ-induced diabetic rats showed a significant increase in the levels of SGPT, SGOT and ALP as compared to the normal control. The Me-Ext, Chf-Ext, and EtAc-Ext (100 and 200 mg/kg) significantly reduced the SGPT, SGOT, and ALP in STZ-induced diabetic rats. The Me-Ext (100 and 200 mg/kg), Chf-Ext and EtAc-Ext (200 mg/kg) also significantly reduced the serum creatinine and blood urea nitrogen in STZ-induced diabetic rats. The standard glibenclamide drug also significantly reduced the SGPT, SGOT, ALP serum creatinine and blood urea nitrogen in STZ-induced diabetic rats.

**Table 6:** Effect of *E. umbellata* fruit extract/fractions on liver and renal functions in streptozotocin-induced diabetic rats

S.No	Treatment groups	Dose (mg/kg)	SGPT (IU)	SGOT (IU)	ALP (IU)	BUN (mg/dl)	Serum creatinine (mg/ml)
1	^a^Normal control	0.3 ml	20 ± 5.6***	16 ± 3.5***	141 ± 7.2**	18.5 ± 3.5**	0.527 ± 0.2***
2	^b^Diabetic control	0.3 ml	62.47 ± 7.5	62.27 ± 6.1	272.57 ± 8.3	35.7 ± 4.5	2.57 ± 0.2
3	^c^Glibenclamide	0.5	24.5 ± 6.4***	20.07 ± 3.6***	142.47 ± 9.3***	17.6 ± 2.3**	0.56 ± 0.2***
4	^c^Me-Ext	100	44.56 ± 6.0*	39.17 ± 3.9**	197.39 ± 10.33*	21.4 ± 5.4**	1.50 ± 0.3*
5	^c^Me-Ext	200	30.37 ± 8.0**	21.97 ± 5.6***	160.19 ± 12.23**	20.3 ± 3.2**	0.85 ± 0.2**
6	^c^Chf-Ext	100	45.70 ± 4.5**	34.37 ± 3.5**	174.22 ± 8.5*	24.6 ± 4.5*	1.46 ± 0.2*
7	^c^Chf-Ext	200	28.80 ± 3.5*	22.37 ± 4.5**	154.32 ± 11.5**	18.6 ± 2.5**	0.76 ± 0.2**
8	^c^EtAC-Ext	100	38.70 ± 4.5*	45.37 ± 2.8*	185.50 ± 17.2*	25.5 ± 5.2*	1.7 ± 0.3*
9	^c^EtAC-Ext	200	28.85 ± 3.5**	25.37 ± 3.5**	165.50 ± 11.2**	21.5 ± 6.2**	1.2 ± 0.2*

Each value is mean ± SEM of 8 animals; comparisons were made between ^a^normal control to ^b^diabetic control using student t-test (** *p* < 0.01, ***p < 0.001) and between ^b^diabetic control to positive control ^c^(Glibenclamide/extract) treated groups using one way ANOVA followed by Dunnett’s posthoc multiple comparison test ((* *p* < 0.05, ** *p* < 0.01, ***p < 0.001)

#### Molecular docking

##### α-Amylase

**Fig. 7:**
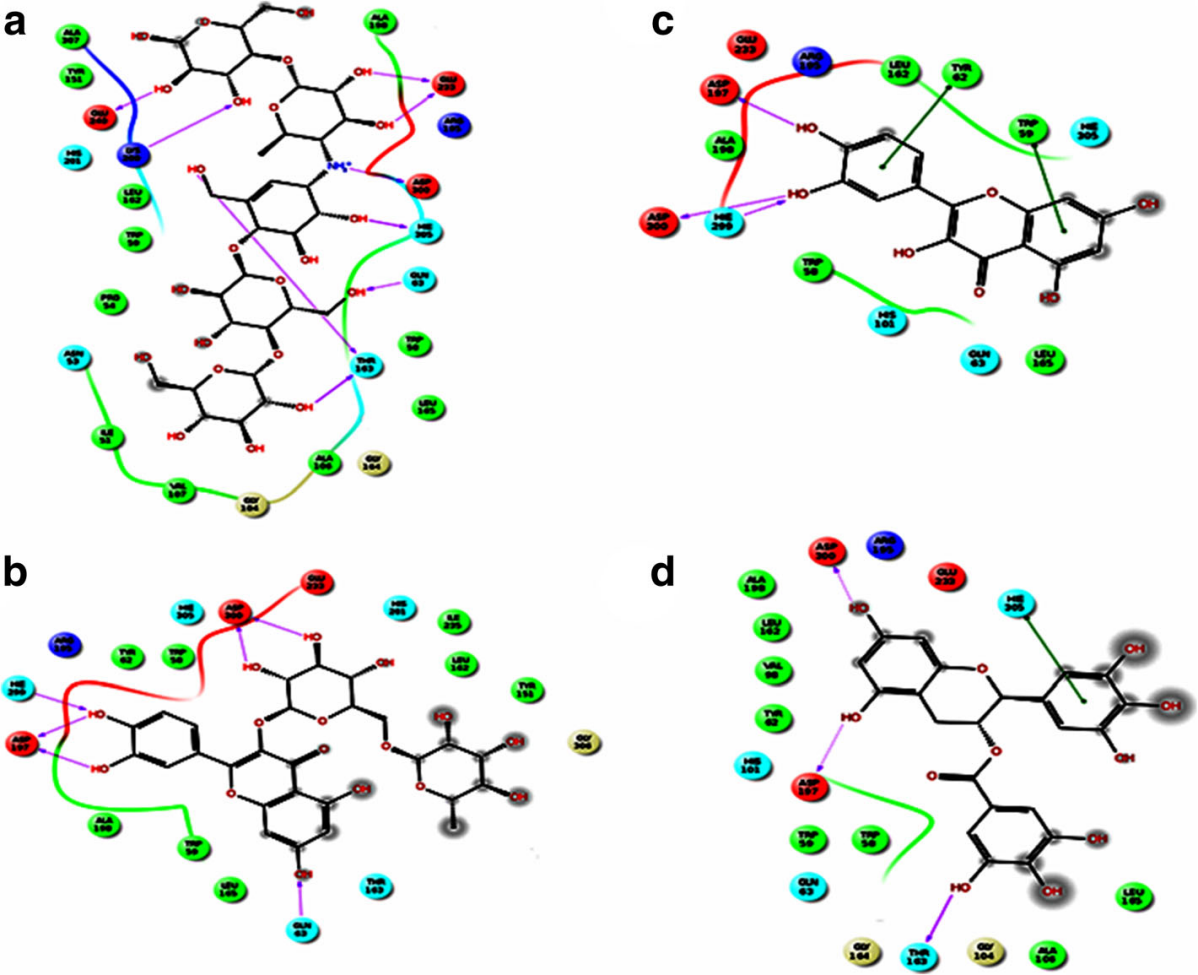
Mode of binding of different compounds and acarbose in α-amylase enzyme active sites. **a** Acarbose, **b** Rutin, **c** Quercetin, and **d** Epigallocatechin gallate

To validate the molecular docking process, the docked acarbose molecule was superimposed to the one obtained from the α-amylase crystal structure, RMSD value of 1.3 Å for all heavy atoms (excluding the hydrogen atoms) was observed. Furthermore, docked acarbose molecule showed similar interactions to those found in the crystal structure [38]. Both the docked acarbose molecule and the crystal structure was shown to be embedded within the binding site and surrounded by a number of hydrophobic residues. In addition, the protonated acarbose amino group was forming an ionic interaction with Asp200. H-bonds were formed with the following amino acid residues Glu240, Lys200, Glu233, and Thr163 (Fig. 7). All docked compounds occupied the same binding site occupied by acarbose. All compounds, except acarbose, occupied a smaller part of the binding site and this ensued in the mixed type to the non-competitive inhibitory effect of these compounds [43, 44]. The GlideScore of all compounds were consistent with the inhibitory activities of α-amylase as shown in Table 7 and were in the order of acarbose, rutin, quercetin, epigallocatechin gallate, epigallocatechin, and catechin hydrate [44, 45]. Figure 7 shows the compounds within the binding site and highlighting the similar interactions with binding site residues. One of the major residues that were found to interact with Asp300. Acarbose was found to form a salt bridge with this residue while all other compounds lacked this interaction and formed H-bonds instead; this tighter interaction would explain the higher inhibitory activity observed for acarbose. In addition, the larger volume occupied by the acarbose molecule in comparison with the smaller compounds may explain the mixed type to the non-competitive inhibitory effect of these compounds [44, 45].

**Table 7:** The GlideScores and IC_50_ values of acarbose and α-amylase inhibitors present in *Elaeagnus umbellata* Thunb

Compound	α-amylase enzyme inhibition (%)^a^	GlideScore
Acarbose	83	−14.158
Rutin	50	−10.434
Quercetin	41	−8.840
Epigallocatechin gallate	21	−7.990
Epigallocatechin	5	−4.550
Catechin hydrate	4	−4.080

Glide Score is an empirical scoring function that estimates the ligand binding free energy, more negative values represent tighter binders. It has been optimized for docking accuracy and binding affinity prediction. Glide Score should be used to rank positions of different ligands in virtual screening. The GlideScore of all compounds were consistent with the inhibitory activities of α-amylase ^a^ [43]

##### α-Glucosidase

The α-glucosidase enzyme crystal structure lacked any ligand within its binding site. In the search for the binding enzyme binding cavity, the Sitemap module [46] was used. Six binding cavities were found; the largest volume cavity was selected for docking so as to accommodate the acarbose large molecule. Subsequently, the grid box was set to be the centroid of the amino acids surrounding this binding site, namely: Arg407, Asp326, Arg197, and Asn258.

**Fig. 8:**
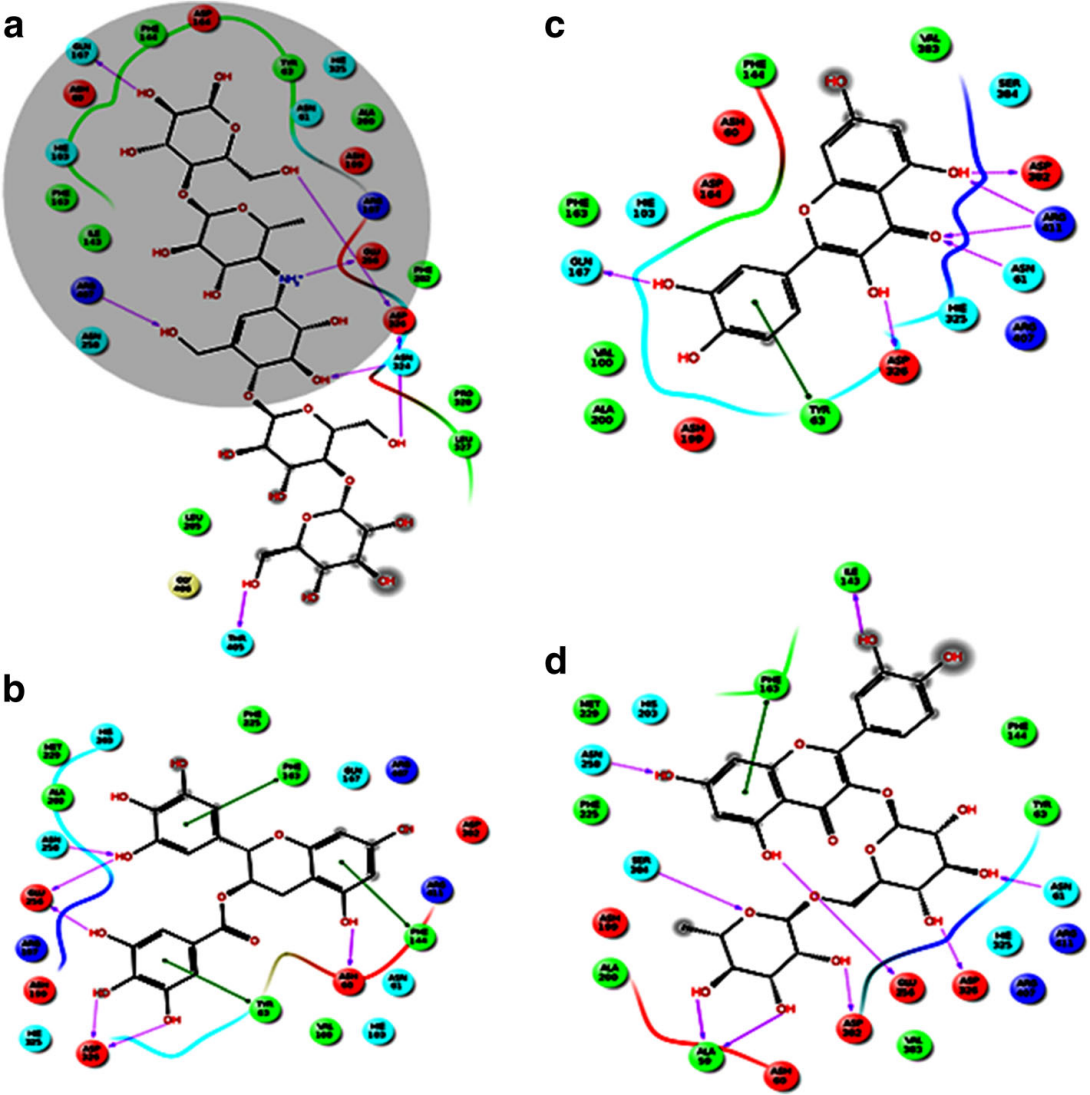
Mode of binding of different compounds and acarbose in α- glucosidase enzyme active sites. **a** Acarbose, **b** Epigallocatechin gallate, **c** Quercetin and **d** Rutin. The highlighted area in **a** is the common area between acarbose and all compounds

All docked compounds only occupied a part of the binding site occupied by acarbose (Fig. 8). Some common interactions were observed between the different binding site residues in acarbose and other compounds used in the docking. Similar to the interactions observed in the α-amylase binding site, the docked test compounds occupied a smaller part of the binding site which also confers the mixed non-competitive inhibitory effect of these compounds on the α-amylase receptor, however, acarbose molecule extended through the full size of the binding site (Fig. 8). The Glide-Scores of all these compounds were found to go in parallel with their experimental α-glucosidase inhibitory activities (Table 8) which followed the order of acarbose, epigallocatechin gallate, quercetin, rutin, epigallocatechin and catechin hydrate [43–45]. It is worth mentioning that all compounds showed weaker inhibitory activity on α-glucosidase than on α-amylase except for epigallocatechin gallate [43, 44].

**Table 8:** The Glide Scores and IC_50_ values of acarbose and α-glucosidase inhibitors present in *Elaeagnus umbellata* Thunb

Compound	α-glucosidae enzyme inhibition (%)^a^	GlideScore
Acarbose	–	−7.725
Epigallocatechin gallate	32	−6.283
Quercetin,	28	−6.258
Rutin	15	−5.830
Epigallocatechin	7	−5.550
Catechin hydrate	1	−4.080

The Glide-Scores of compounds: acarbose, epigallocatechin gallate, quercetin, rutin, epigallocatechin and catechin hydrate were found to go in parallel with their experimental α-glucosidase inhibitory activities. All compounds showed weaker inhibitory activity on α-glucosidase except for epigallocatechin gallate. The more negative Glide Score values represent tighter binders ^a^ [43]

## Discussion

In spite of the available antidiabetic medications, herbal remedies and extracts are of great importance for the ethnobotanical community and are considered to be less toxic than the synthetic drugs [47]. There has been an increasing interest of the scientific community in the traditional and herbal medicines due to their pharmacological and economic advantages [48]. Medicinal plants received much attention due to the existence of indispensable bioactive compounds such as phenolics and flavonoids which shown strong antioxidant property [49, 50].

The objective of the current investigation was to comprehensively evaluate the antioxidant and antidiabetic potential of *E. umbellata* fruit as an indigenous medicinal plant. In the current study, the Me-Exts of *E. umbellata* fruit and their subsequent fractions showed strong antioxidant potential, which might be due to the presence of phenolic and flavonoid compounds. HPLC-UV fingerprints of the Me-Ext and subsequent fractions of *E. umbellata* fruit also confirmed the presence of phenolic acid and flavonoid compounds, which is an agreement with a previously reported study showing the presence of phenolic acids (gallic acid, vanillic acid, coumaric acid, sinapic acid, ferulic acid and caffeic acids) in the hydro methanolic berry extracts [51].

Significant antioxidant potential was exhibited by extracts/fractions of *E. umbellata* fruit against DPPH and ABTS. The results of the current study revealed that the highest % radical scavenging potential was exhibited by Chf-Ext and EtAc-Ext fraction. % DPPH and ABTS inhibition potential of various plant test samples were comparable with standard ascorbic acid (positive control) showing a concentration-dependent response.

α-Amylase is the key enzyme in the human body that is responsible for the breaking down of polysaccharides starch into disaccharides. The α-glucosidase enzyme causes the hydrolysis of disaccharides into simple sugars which are subsequently absorbed through small intestine thus causing postprandial hyperglycemia [52]. Thus α-Amylase inhibitors prevent the absorption of dietary starch, and decrease the postprandial glucose level. Inhibiting the breakdown of starch may have useful effects in diabetic people [53]. In our study, we found that the Me-Ext, Chf-Ext and EtAc-Ext fractions of *E. umbellata* significantly inhibited α-amylase and α-glucosidase enzymes indicating antihyperglycemic effects. The IC_50_ values of Chf-Ext and EtAc-Ext were found comparable with the positive control (acarbose) showing a concentration-dependent response. These data suggest that the antidiabetic agents are preferentially present in these extracts.

Furthermore, validation of molecular docking procedure, the superimposition of docked acarbose molecule to the one that has obtained from the α-amylase crystal structure and similar interactions were found in the α-amylase binding site. The comparable binding modes of all molecules within the vicinity of the acarbose binding site emphasized that the effects of Chf-Ext and EtAc-Ext are due to their organic constituents.

The use of HFD and STZ to induce T2D in rats has already been reported in the literature [33, 34, 54]. In this model, an administration of HFD causes obesity in rats which leads to insulin resistance. Furthermore, a low dose of STZ which is known as diabetogenic and a β-cell toxin causes destruction and severe decline of β-cells [55, 56]. As a result, the lack of insulin causes hyperglycemia [57]. Thus the hyperglycemia coupled with other metabolic irregularities including insulin resistance and hyperlipidemia closely depicts the metabolic appearances of T2D in humans [58, 59]. Furthermore, in normal metabolic condition, insulin causes lipid metabolism through activation of a lipoprotein-lipase enzyme that breaks down triglycerides to fatty acids and glycerol. These fatty acids are used as energy or re-esterified in the body tissues for storage. In T2D insulin insufficiency or resistance leads to inactivation of lipoprotein lipase causes a condition of hypertriglyceridemia. In this study, the major changes in lipid profile such as a high serum triglycerides, serum cholesterol, serum LDL cholesterol and low serum HDL cholesterol in STZ- induced diabetic rats are in agreement with the lipid profiles alterations reported by other researchers [7, 48]. High LDL level is characterized by transporting cholesterol to the tissues from the liver that leads to the development of coronary heart disease [60]. While, HDL cholesterol is considered as a valuable lipoprotein that transport endogenous cholesterol and cholesteryl esters to the liver and steroidogenic tissues from the body tissues and prevent deposition of cholesterol, thus inhibiting atherosclerosis [61].

In the current study, the extracts/fractions of *E. umbellata* significantly reduced blood glucose in STZ (50 mg/kg) induced diabetogenic animal model. This reduction in blood glucose level by *E. umbellata* fruit extract/fractions might be due to the inhibition of STZ induced free radicals by phenolic and flavonoid compounds present in *E. umbellata.* The antihyperglycemic effect of Me-Ext and Chf-Ext of *E. umbellata* was equivalent to standard glibenclamide. Furthermore, the phytoconstituents present in the Me-Ext, Chf-Ext, and EtAc-Ext may increase the secretion of insulin from pancreatic βeta-cells, thus resulting in an improvement in glycemic control.

Weight loss is also a serious problem in STZ induced diabetes which may be due to hyperglycemia, hypoinsulinemia, loss of proteins and muscle wasting [62]. STZ-induced diabetic rats revealed a significant reduction in the body weight as compared to the normal control rats during the experimental study period. The extract/fractions of *E. umbellata* significantly increase the STZ mediated reduction in body weight. This outcome is consistent with previous studies and could be due to the capability *E. umbellata* extracts to reduce hyperglycemia [62–64]. Moreover, the Me-Ext, Chf-Ext, and EtAc-Ext showed a significant decrease in TC, TGs, LDL and cholesterol while significantly increased HDL cholesterol in the diabetic control group at the end of the experiment.

Studies have shown that STZ induces CYP2E1 dependent oxidative stress and causes the release of various liver microsomal enzymes including SGOT, SGPT and serum ALP in the blood that indicates liver damage or condition of T2D disease [65, 66]. The extracts/fractions of *E. umbellata* have significantly reduced the levels of SGPT, SGOT, and ALP in the diabetic control group that indicates a possible hepatoprotective effect. The standard glibenclamide drug also significantly reduced the levels of SGPT, SGOT, and ALP in the diabetic control group. Furthermore, the Me-Ext, Chf-Ext, and EtAc-Ext also caused a significant reduction in serum creatinine and blood urea nitrogen indicating protective effects on kidneys.

Thus it is possible that the phenolic and flavonoid compounds present in these extracts may act against the oxidative stress-related hepatotoxicity produced by the induction of CYP2E1 in STZ-induced diabetes and thereby protect the liver [67]. STZ-induced diabetes is usually associated with impairment of renal function as mediated by significant increases in serum creatinine level and blood urea nitrogen. This is due to the interaction of STZ with glomerular tissues and glomerular filtrations [68]. In the current study, the Me-Ext, Chf-Ext, EtAc-Ext, and standard antidiabetic drug glibenclamide also significantly reduced the serum creatinine level and blood urea nitrogen in diabetic control rats that revealed its renoprotective effect.

The overall antidiabetic activity of the Me-Ext and subfractions of *E. umbellata* may be due to their strong antioxidant potential. In addition to reducing carbohydrate metabolism by inhibiting α-amylase and α-glucosidase enzymes, the phenolic and flavonoids compounds may exert an antidiabetic effect by decreasing the intestinal carbohydrate absorption, increasing insulin action or insulin secretion, increase in β-cell function and antioxidant effect [69].

These data confirmed that the Me-Ext and subfractions of *E. umbellata* have significant antidiabetic activity against α- glucosidase and α- amylase enzymes in STZ-induced diabetes mellitus supported by docking analysis. Furthermore, these extracts have protective effects on the major tissues including liver and kidney and thus reduce diabetes-associated complications.

To the best of our knowledge, this is the first study reporting the antidiabetic activity of *E. umbellata* (silver berry) fruits/berry. However, this study is limited to the in vitro and in vivo evaluation of antidiabetic effects of the crude extract and their fractions. Further studies are required to isolate the phytoconstituents responsible for the antidiabetic activity and to elucidate their mechanism of action including effects on various specific markers of Diabetes mellitus including insulin and glycated hemoglobin levels.

## Conclusion

In conclusion, the Me-Ext their subsequent fractions like Chf-Ext and EtAc-Ext of *E. umbellata* fruits/berries significantly reduced blood glucose levels in in-vitro studies as well as in vivo in high-fat diet (HFD) and low dose STZ-induced diabetic rats. These extracts also showed hypolipidemia, hepatoprotective and nephroprotective effects. These effects might be due to the presence of phenolic and flavonoids phytoconstituents present in these extract/fractions.

## Additional files

Additional file 1: **Figure S1.** *E. umbellata* Thunb. (Autumn Olive). a) *E. umbellata* Thunb. Tree b) *E. umbellata* Thunb. red berried shrubs c) *E. umbellata* Thunb. berries/ fruits. (TIF 700 kb)

Additional file 2: **Table S1.** Effect of *E. umbellata* fruit methanolic extract/fractions on blood glucose level in streptozotocin induced diabetic rats. Each value is mean ± SEM of 8 animals. Comparisons were made between ^a^normal control to ^b^diabetic control using student t-test (***p < 0.001) and between ^b^diabetic control to positive control ^c^(Glibenclamide/extracts) treated groups using one way ANOVA followed by Dunnett’s posthoc multiple comparison test (* *p* < 0.05,** *p* < 0.01, ***p < 0.001). (DOCX 16 kb)

Additional file 3: **Table S2.** Effects of *E. umbellata* fruit methanolic extract/fractions on body weight in STZ-induced diabetic rats. Each value is mean ± SEM of 8 animals. Comparisons were made between ^a^normal control to ^b^diabetic control using student t-test (***p < 0.001) and between ^b^diabetic control to positive control ^c^(Glibenclamide/extracts) treated groups using one way ANOVA followed by Dunnett’s posthoc multiple comparison test (* *p* < 0.05,** *p* < 0.01). % change in body weight = initial weight (g) - final weight / initial weight (g) × 100. (DOCX 16 kb)

## Abbreviations

ABTS: 2, 2′-azinobis-3-ethylbenzothiazoline-6-sulfonic acid
AD: Alzheimer’s disease
ALP: Serum alkaline phosphatase
Aq.Ext: Aqueous extract fraction
But.Ext: *n*-Butanol extract fraction
Chf-Ext: Chloroform extract fraction
DM: Diabetes mellitus
DNSA: 3, 5-dinitrosalicylic acid
DPPH: 2, 20-diphenyl-1-picrylhydrazyl
EtAc-Ext: Ethyl acetate extract fraction
HDL: High density lipoprotein
Hex.Ext: *n*-hexane extract fraction
HFD: High-fat diet
HPLC-UV: High performance liquid chromatography- Ultraviolet
IC_50_: Median inhibitory concentration
LDL: Low density lipoproteins
Me-Ext: Hydromethanolic extract
ROS: Reactive oxygen species
Rt: Retention time
SEM: Standard error mean
SGOT: Serum glutamate oxaloacetate transaminase
SGPT: Serum glutamate pyruvate transaminase
STZ: Streptozotocin
T2D: Type 2 diabetes
TC: Total cholesterol
TG: Triglycerides
UVAD: Ultraviolet array detector
