# Supplementary figures and images for: Phytochemical analysis and antidiabetic potential of Elaeagnus umbellata (Thunb.) in streptozotocin-induced diabetic rats: pharmacological and computational approach
Source: BMC Complement Altern Med. 2018 Dec 13;18:332. doi: 10.1186/s12906-018-2381-8 (PMC6293591; doi:10.1186/s12906-018-2381-8)

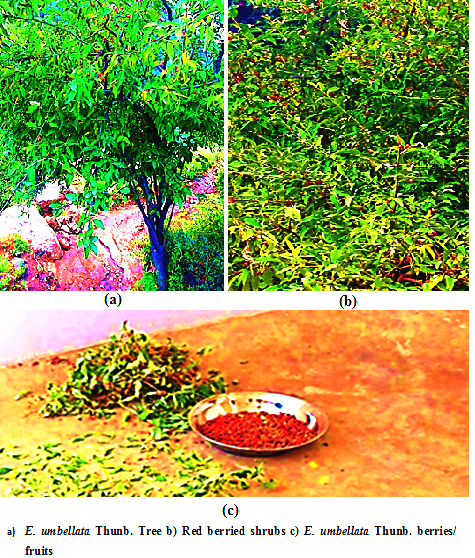

Supplement: Supplementary file 1 — Figure S1. E. umbellata Thunb. (Autumn Olive). a) E. umbellata Thunb. Tree b) E. umbellata Thunb. red berried shrubs c) E. umbellata Thunb. berries/ fruits. (TIF 700 kb) [file 12906_2018_2381_MOESM1_ESM.tif]
