# Supplementary material for: Phytochemical analysis and antidiabetic potential of Elaeagnus umbellata (Thunb.) in streptozotocin-induced diabetic rats: pharmacological and computational approach
Source: BMC Complement Altern Med. 2018 Dec 13;18:332. doi: 10.1186/s12906-018-2381-8 (PMC6293591; doi:10.1186/s12906-018-2381-8)
Supplement: Supplementary file 2 — Table S1. Effect of E. umbellata fruit methanolic extract/fractions on blood glucose level in streptozotocin induced diabetic rats. Each value is mean ± SEM of 8 animals. Comparisons were made between anormal control to bdiabetic control using student t-test (***p < 0.001) and between bdiabetic control to positive control c(Glibenclamide/extracts) treated groups using one way ANOVA followed by Dunnett’s posthoc multiple comparison test (* p < 0.05,** p < 0.01, ***p < 0.001). (DOCX 16 kb) [file 12906_2018_2381_MOESM2_ESM.docx]

**Table S1 Effect of *E. umbellata* fruit methanolic extract/fractions on blood glucose level in streptozotocin induced diabetic rats**

| **S.No** | **Groups** | **Dose (mg/kg)** | **1^st^ day** | **5^th^ day** | **8^th^ day** | **10^th^ day** | **15^th^ day** | **21^st^ day** |
| --- | --- | --- | --- | --- | --- | --- | --- | --- |
| 1 | Normal control | 0.3ml | 103± 22 | 110±13*** | 108±12******* | 102±11******* | 109±10******* | 104±15******* |
| 2 | Diabetic control | 0.3ml | 371±37 | 344±30 | 380.87±50 | 376±34 | 316±50 | 384±30 |
| 3 | Glibenclamide | 0.5 | 364±21 | 203±43****** | 207±35****** | 201±22****** | 152±31******* | 136±21******* |
| 4 | ^c^Me-Ext | 100 | 361±43 | 273±54* | 260±87* | 240±67** | 222±12** | 150±32*** |
| 5 | ^c^Me-Ext | 200 | 370±23 | 286±45* | 245±14* | 210±21** | 164±10** | 140±20*** |
| 6 | ^c^Chf-Ext | 100 | 372±19 | 285±13* | 280±23* | 255±15* | 210±21** | 168±18** |
| 7 | ^c^Chf-Ext | 200 | 374±21 | 281±32* | 260±33* | 245±20** | 190±31** | 138±22*** |
| 8 | ^c^EtAC-Ext | 100 | 365±17 | 340±14^ns^ | 365±17 ^ns^ | 356±14 ^ns^ | 353±19 ^ns^ | 333±22* |
| 9 | ^c^EtAC-Ext | 200 | 361±17 | 339±12* | 325±37* | 286±24* | 233±29* | 193±12* |

Each value is mean ± SEM of 8 animals. Comparisons were made between ^a^normal control to ^b^diabetic control using student t-test (***p < 0.001) and between ^b^diabetic control to positive control ^c^(Glibenclamide/extracts) treated groups using one way ANOVA followed by Dunnett’s posthoc multiple comparison test (* p < 0.05,** p < 0.01, ***p < 0.001)
