# Supplementary material for: Phytochemical analysis and antidiabetic potential of Elaeagnus umbellata (Thunb.) in streptozotocin-induced diabetic rats: pharmacological and computational approach
Source: BMC Complement Altern Med. 2018 Dec 13;18:332. doi: 10.1186/s12906-018-2381-8 (PMC6293591; doi:10.1186/s12906-018-2381-8)
Supplement: Supplementary file 3 — Table S2. Effects of E. umbellata fruit methanolic extract/fractions on body weight in STZ-induced diabetic rats. Each value is mean ± SEM of 8 animals. Comparisons were made between anormal control to bdiabetic control using student t-test (***p < 0.001) and between bdiabetic control to positive control c(Glibenclamide/extracts) treated groups using one way ANOVA followed by Dunnett’s posthoc multiple comparison test (* p < 0.05,** p < 0.01). % change in body weight = initial weight (g) - final weight / initial weight (g) × 100. (DOCX 16 kb) [file 12906_2018_2381_MOESM3_ESM.docx]

**Table S2 Effects of *E. umbellata* fruit methanolic extract/fractions on body weight in STZ-induced diabetic rats**

| **S.No** | **Groups** | **Dose (mg/kg)** | **1^st^ day** | **5^th^ day** | **8^th^ day** | **10^th^ day** | **15^th^ day** | **21^st^ day** | **% change in b.w (g)** |
| --- | --- | --- | --- | --- | --- | --- | --- | --- | --- |
| 1 | ^a^Normal control | 0.3ml | 164±2 | 163±3 | 169±1 | 170±2 | 172±3 | 173±2*** | +5.4 |
| 2 | ^b^Diabetic control | 0.3ml | 167±2 | 164±7 | 147±4 | 139±05 | 131±4 | 130±2 | -22.15 |
| 3 | ^c^Glibenclamide | 0.5 | 168±3 | 172±3 | 185±3 | 185±12 | 190±6 | 189±4** | +12.5 |
| 4 | ^c^Me-Ext | 100 | 167±2 | 171±2 | 174±2 | 179±4 | 181±6 | 178±3** | +6.5 |
| 5 | ^c^Me-Ext | 200 | 170±2 | 170±7 | 180±3 | 184±3 | 190±4 | 190±4** | +11.7 |
| 6 | ^c^Chf-Ext | 100 | 168±3 | 169±3 | 170±2 | 173±4 | 173±2 | 175±3** | +4.1 |
| 7 | ^c^Chf-Ext | 200 | 166±2 | 167±2 | 171±4 | 172±7 | 175±3 | 188±2** | +13.2 |
| 8 | ^c^EtAC-Ext | 100 | 165±3 | 166±4 | 157±2 | 146±3 | 158±4 | 169±4* | +2.4 |
| 9 | ^c^EtAC-Ext | 200 | 168±3 | 168±2 | 175±3 | 171±5 | 171±7 | 179±3** | +6.5 |

Each value is mean ± SEM of 8 animals. Comparisons were made between ^a^normal control to ^b^diabetic control using student t-test (***p < 0.001) and between ^b^diabetic control to positive control ^c^(Glibenclamide/extracts) treated groups using one way ANOVA followed by Dunnett’s posthoc multiple comparison test (* p < 0.05,** p < 0.01)

$$\% change in body weight = initial weight (g) - final weight \boldsymbol{/} initial weight (g) \times100$$
